# Continuing Education Resources and Tools for the Advanced Practitione

**Published:** 2012-09-01

**Authors:** Wendy Vogel

**Affiliations:** Ms. Vogel is an oncology nurse practitioner from Wellmont Cancer Institute in Kingsport, Tennessee.

## Abstract

Are you looking for some continuing education (CE) credits? Maybe you want to learn about a certain topic? Visit www.advancedpractitioner.com to access JADPRO’s current CE offerings, but take advantage of the great (mostly free) resources listed here as well. Then sit down and get ready to learn!

## Clinical Care Options

www.clinicaloptions.com

Clinical Care Options provides free, interactive medical education programs for health-care professionals in areas such as oncology, transplantation, urology, HIV, and hepatitis. Educational opportunities translate the latest developments in science and medicine to clinically useful information. Content is offered simultaneously online, in print, and via live meeting and conference coverage. Downloadable slide sets, worksheets, and support tools are available. 

**Cost: **Free

## CME LLC

www.cmellc.com/oncology/

CME LLC provides free continuing education to health-care professionals in the areas of oncology, primary care, and psychiatry. Educational plans are designed to meet the needs of practicing clinicians based on demonstrated gaps within a target audience. All educational offerings are reviewed by an independent and blinded three-tier peer review process. Students can learn through case studies, multimedia activities, and journals such as the *ONCOLOGY Nurse Edition* and *Gastrointestinal Cancer Research.*

**Cost: **Free

## Epocrates

www.epocrates.com

Epocrates is a Web- and smartphone-based application that provides "point-of-service" information. The smartphone application provides an interactive drug database, medical calculators, a drug interaction calculator, a pill photo identifier, and more, including mobile CME. There is a free version of Epocrates that can be upgraded with enhanced features for a yearly fee; CME is free. The mobile version requires an Internet connection to complete mobile activities (only Android or iPhone operating systems), but an online version is also available. Hematology/oncology-specific content is available, and the user can create a personalized learning plan based on his/her areas of interest.

**Cost: **CME is free; many other features are free but some require a yearly subscription. 

## Institute for Medical Education and Research (IMER)

www.imeronline.com

IMER is a free Web-based site that offers health-care professionals fair-balanced, scientifically rigorous, expert-led accredited educational programs designed to foster professional development. It provides CME through video presentations, monographs, and other educational activities. IMER also offers education presented at national and regional professional meetings. Live presentations are offered and may be requested for your facility. Professional resources such as mechanism of action videos, a multiple myeloma mentorship program, and nurse educator partnerships are available. Clinical tools for staging, drug interactions, chemotherapy calendar, screening and assessment, ASCO updates, and more will assist with at-the-bedside care. Links to other valuable resources are also provided.

**Cost: **Free

## Medscape

www.medscape.com

Medscape is a free online resource for specialists, primary care practitioners, pharmacists, and other health-care professionals that provides integrated medical information and educational tools. You may choose a personalized specialty site homepage, including Oncology/Hematology or Advanced Practice. Medscape offers original, professional medical content, including review articles, journal commentary, expert columns, patient education articles, book reviews, and more. You can find conference coverage, professional medical news coverage, access to MEDLINE, access to medical journals and textbooks, business practice information, and CME. 

**Cost: **Free

## Meniscus Educational Institute

www.mensicus.com

Meniscus provides free CE for physicians, pharmacists, advanced practitioners, and nurses. Meniscus collaborates with health-care professionals, communities, foundations, and organizations to provide medical education that will benefit patient care. Primary care and oncology offerings are available. Choose a "learning channel" (such as oncology, colorectal cancer, and latest oncology news channels) for a unique educational experience. Surveys online assist with determining educational needs.

**Cost: **Free

## QuantiaMD

www.quantiamd.com

QuantiaMD is a free online community where practicing clinicians share practical medicine. Despite the name of the site, advanced practitioners are also welcome. This innovative site provides an opportunity for social learning by way of short real-world cases delivered by real-world clinicians. Sessions are short and interactive, usually no longer than 5 minutes, although topics that offer CME are longer. QuantiaMD offers both categories 1 and 2 CME. There is an oncology community with oncology/hematology-specific presentations. Other medical communities include pain, cardiovascular, diabetes, infectious disease & HIV, mental health, professional practice, aging, patient relationships, and more. QuantiaMD is accessible via computer, smartphone or iPad. One perk associated with this site is the ability to earn Amazon dollars by participating in engaging on-line activities!

**Cost: **Free

## *The Oncologist* CME Online

http://cme.alphamedpress.org/

*The Oncologist*, published by AlphaMed Press, is a free peer-reviewed journal dedicated to translating the latest oncology research developments into best multimodality practice. The emphasis of the journal is on clear, concise interpretation, rather than data, and its impact on oncology practice. CME is available through the journal, video, and mobile applications (iPad, iPhone, Android, and Kindle Fire). The free CME is approved for category 1 credit. Free registration is required.

**Cost: **Free

## UpToDate

www.uptodate.com

UpToDate is a decision support system that helps find answers to clinical questions quickly and easily at the point of care. Many health-care facilities subscribe to this system for their employees. By creating your own account and logging in with each use, "effortless" CME is earned with every search just for looking up answers/information you need on the job! Access is available through the Web and mobile devices (iPhone and iPad). Oncology/hematology-specific content and patient education resources are available.

**Cost: **A 1-year personal subscription is $499, although 30-day increments may be purchased for $45.

